# Impacts of Resveratrol and Pyrogallol on Physicochemical, Mechanical and Biological Properties of Epoxy-Resin Sealers

**DOI:** 10.3390/bioengineering9030085

**Published:** 2022-02-22

**Authors:** Naji Kharouf, Salvatore Sauro, Louis Hardan, Amr Fawzi, Ilona Eveline Suhanda, Jihed Zghal, Frédéric Addiego, Christine Affolter-Zbaraszczuk, Youri Arntz, Vincent Ball, Florent Meyer, Youssef Haikel, Davide Mancino

**Affiliations:** 1 Department of Endodontics and Conservative Dentistry, Faculty of Dental Medicine, Université de Strasbourg, 67000 Strasbourg, France; ilona-eveline.suhanda@etu.unistra.fr (I.E.S.); youri.arntz@unistra.fr (Y.A.); vball@unistra.fr (V.B.); fmeyer@unistra.fr (F.M.); youssef.haikel@unistra.fr (Y.H.); mancino@unistra.fr (D.M.); 2 Institut National de la Santé et de la Recherche Médicale, INSERM UMR_S 1121 Biomaterials and Bioengineering, 67085 Strasbourg, France; c.affolter-zbaraszczuk@unistra.fr; 3 Dental Biomaterials and Minimally Invasive Dentistry, Department of Dentistry, Cardenal Herrera-CEU University, CEU Universities, C/Santiago Ramón y Cajal, s/n., Alfara del Patriarca, 46115 Valencia, Spain; salvatore.sauro@uchceu.es; 4 Department of Therapeutic Dentistry, I. M. Sechenov First Moscow State Medical University, 119146 Moscow, Russia; 5 Department of Restorative Dentistry, Saint-Joseph University, Beirut 11072180, Lebanon; louis.hardan@usj.edu.lb; 6 UWA Dental School, University of Western Australia, Nedlands, WA 6009, Australia; amr.fawzy@uwa.edu.au; 7 ICube Laboratory, UMR 7357 CNRS, Mechanics Department, University of Strasbourg, 67000 Strasbourg, France; zghal@unistra.fr; 8 Laboratoire Energetique Mecanique Electromagnetisme, University of Paris Ouest, 50 rue de Sèvres, 92410 Ville d’Avray, France; 9 Luxembourg Institute of Science and Technology (LIST), Department Materials Research and Technology (MRT), ZAE Robert Steichen, 5 rue Bommel, L-4940 Hautcharage, Luxembourg; frederic.addiego@list.lu; 10 Pôle de Médecine et Chirurgie Bucco-Dentaire, Hôpital Civil, Hôpitaux Universitaire de Strasbourg, 67000 Strasbourg, France

**Keywords:** endodontic sealer, polyphenols, resveratrol, pyrogallol, biological activity

## Abstract

This study aimed at evaluating the physicochemical and biological properties of experimental epoxy-resin sealers containing polyphenols such as resveratrol and pyrogallol. A conventional epoxy resin (OB) was modified by adding different concentrations of resveratrol (RS) or pyrogallol (PY) to its composition. Antibacterial and antioxidant activities, mechanical properties, along with wettability and morphological changes were investigated. The results were statistically analyzed using ANOVA and multiple comparison tests (α = 0.05). The incorporation of the tested polyphenols into the epoxy resin enhanced its mechanical properties. PY demonstrated much better antioxidant and antibacterial activities than RS, which were associated with a higher release of PY. In contrast, PY showed a higher cytotoxicity than OB and OB doped with RS. OB containing PY presented a rougher surface and higher water absorption than OB doped with RS. Both tested polyphenols caused no notable changes to the overall porosity of OB. Resveratrol and pyrogallol may not only influence the morphology and mechanical properties of epoxy-resin sealers, but could also enhance antioxidant activity and antibacterial effects against *Enterococcus faecalis*. Most epoxy-resin sealers currently available in the market can be considered as “passive” materials. Thus, doping their composition with specific polyphenols may be a suitable strategy to confer some antibacterial properties, antioxidant potential, along with improvement of some mechanical properties.

## 1. Introduction

For appropriate preparation of a cavity access, good shaping, proper cleaning and well-sealed tridimensional filling of the root canal space are essential factors in order to achieve a successful endodontic treatment [1,2]. Since it does not seem possible to entirely eliminate bacteria in an infected root canal system, a clinician should try to entomb such residual bacteria during obturation procedures, along with their byproducts, as much as possible. Indeed, to provide a hermetic tridimensional seal of the root canal system, gutta-percha is used in combination with root canal sealers during endodontics procedures [2,3].

Nevertheless, the incidence of voids formation during such procedures is still very high and proliferation of residual bacteria in such circumstances is inevitable; the risk for failure in the long-term outcome of root canal treatments is highly plausible [1,2,3]. A root canal sealer should be characterized as having good sealing ability, exhibiting low solubility, along with having high biocompatibility and antibacterial activity [3,4]. Depending on their chemical compositions, several types of root canal sealers are currently available in clinics, such as glass ionomer cements, silicone-based sealers, epoxy resins, calcium silicate-based sealers and zinc oxide-eugenol [3,5]. Calcium silicate-based sealers demonstrate good filling ability and antibacterial activity [3,6] but they are still overly expensive [7]. Conversely, epoxy resin-based sealers are relatively inexpensive, but they are characterized by limited or no antibacterial activity [8,9,10] especially against *Enterococcus faecalis* (*E. faecalis*), a predominant bacteria found in persistent endodontic infections and root canal re-treatment cases [3,8].

Polyphenols have been used to modify various dental materials in order to enhance their mechanical properties, biological activity and bonding strength to tooth tissues [11]. For instance, Hu et al. [12] used epigallocatechin-3-gallate (EGCG) in glass ionomer cements to improve their antibacterial ability, along with flexural strength and surface micro-hardness. In a further study [4] a cement with a mineral trioxide aggregate (MTA) base was doped with tannic acid (TA); there was an increase in compressive stiffness at higher concentrations of TA. Resveratrol (RS) (3,4,5-trihydroxystylbene), a polyphenol from the stilbene family characterized by a phytoalexin phenolic compound, was incorporated into dentin bonding agents in order to improve their biocompatibility [13]. RS has been used to treat dentin before the application of dental adhesives in order to preserve the dentin-resin bond over time [14]. Moreover, pyrogallol (PY) (1,2,3-trihydroxybenzene) is a well-known organic compound produced in plants [15] that is often used for its potent antibacterial activity against Staphylococcus aureus [16]. PY has been used in endosseous implant applications as a nanocoating; the release of PY can reduce significantly the growth of planktonic bacteria [17]. Moreover, the presence of pyrogallol groups in tannic acid have been demonstrated to play a role in the treatment of dentin hypersensitivity [18]. Several studies reported that resveratrol and pyrogallol exhibit antibacterial activity against Gram- and Gram+ bacteria [16,19,20,21].

Since epoxy-resin sealers have no evident antibacterial activity, the main idea of the present study was to incorporate some specific polyphenols with antibacterial properties into a conventional sealer currently available in the market. Thus, the purpose of the present study was to evaluate morphological changes, antibacterial activity, cytotoxicity and chemical-mechanical properties of an epoxy-resin endodontic sealer doped with different polyphenols, such as pyrogallol and resveratrol. The null hypothesis was that the addition of the different polyphenols (RS or PY) would have no impact on physicochemical and biological properties of tested epoxy-resin sealers.

## 2. Materials and Methods

### 2.1. Experimental and Control Materials

A conventional epoxy-resin dental sealer currently available in the market (OB) (Obturys, ITENA Clinical, Paris, France) was used in this study and labelled as the control group (Table 1). It was also used to generate several experimental cements by mixing different concentrations (0.5 wt%, 1 wt% and 5 wt%) of pyrogallol “PY” (Sigma Aldrich, Saint-Quentin-Fallavier, France, ref. P0381) or resveratrol “RS” (Sigma Aldrich, Saint-Quentin-Fallavier, France, ref. R5010). It was not possible to incorporate more than 5% of RS or PY to prepare such experimental materials as a result of issues related to mixing, which caused inhomogeneous blends on a visual basis.

### 2.2. Specimen Preparations

The epoxy-resin dental sealer (OB) used in this study has two components (base and catalyst), but the polyphenols (RS or PY) were only added to the base part of the sealer and mixed using a mixing spatula for approximately 1 min until a homogenous paste was obtained on a visual basis. Subsequently, the complex (powder + base) was added to the catalyst paste and mixed for 1 min in order to obtain the experimental PY@OB or RS@OB sealers. All groups of specimens were labelled as OB (control group, epoxy resin without the addition of polyphenols), OB@RS (epoxy-resin modified with resveratrol) and OB@PY (epoxy-resin modified with pyrogallol). Stainless steel ring molds (internal diameter 20 ± 0.1 mm and height 1.6 ± 0.1 mm) were used to prepare the specimens for solubility, contact angle and roughness tests. Meanwhile, discs of 10 mm in diameter and 2 mm in height were prepared to test the release kinetic of the polyphenols and to perform surface ultra-morphology analysis via scanning electron microscopy. Finally, cylindrical specimens (internal diameter 3 mm and height 3.8 mm) were prepared for evaluation of their compressive strength, porosity, cytotoxicity and antioxidant properties. All specimens were stored in the dark in a container at 37 °C for 48 h in order to achieve a proper setting time.

### 2.3. Antimicrobial Activity

#### 2.3.1. Bacterial Strain

*Enterococcus faecalis* (*E. faecalis*, ATCC 29212) was cultured in Brain Heart Infusion medium (BHI) (Darmstadt, Germany). In all tests, the turbidity of BHI containing *E. faecalis* was adjusted to OD_600 (nm)_ = 0.3.

#### 2.3.2. Agar Diffusion Tests (ADT)

The ADT tests were performed as described in previous studies [3]. In brief, six agar-filled petri dishes each containing 25 mL of BHI agar were used to evaluate antibacterial activity of the experimental materials OB@RS and/or OB@PY at different concentrations of PY and RS (0.5 wt%, 1 wt% and 5 wt%). A precise amount of the bacterial medium (100 µL) was spread homogeneously onto the petri dishes. Four wells in each petri dish, 3.0 mm in diameter and 3.0 mm in depth, were made with an adapted whack (3 mm, PFM medical, Köln, Germany) by removing the agar. The first three wells were filled with 0.5, 1 and 5 wt% of OB@PY or OB@RS, while the fourth well was filled with the control group (OB). All the agar petri dishes were incubated at 37 °C for 24 h. The inhibition zone in each group was assessed after 24 h of incubation [3].

#### 2.3.3. Direct Contact Tests (DCT)

Immediately after mixing, for each tested group (in triplicate), each composite was placed (0.06 g per well) in the center of each well (24-well culture plates) (Trefflab, Degersheim, Switzerland). A specific amount of *E. faecalis* (1 mL) was added to each well and incubated for 24 h anaerobically at 37 °C under constant stirring (450 rpm). Positive control was set as *E. faecalis* culture without material. At each time step, the *E. faecalis* concentration was measured by manual counting. Briefly, 10-fold serial dilutions up to 10^5^ in BHI were performed on each specimen of *E. faecalis* solution. Onto a BHI agar plate was homogeneously spread 100 μL of each dilution, and then incubated at 37 °C. After 24 h of incubation, colonies on the plate were counted and their CFU/mL (colony forming units/mL) were determined from the dilution plate.

### 2.4. Cytotoxicity Test

NIH3T3 cell line (mouse fibroblast ATCC CRL-1658) was cultivated in high-glucose content Dulbecco’s Modified Eagle Medium (DMEM) with 10% fetal bovine serum and 1% of penicillin-streptomycin (Dominique Dutscher, Bernolsheim, France). Activated media were prepared by incubating OB, OB@5%RS and OB@5%PY specimens (in triplicate) in 500 µL of complete medium for 24 h at 37 °C. Prior to the experiment, NIH3T3 were seeded in a 96-well plate at a concentration of 8000 cells per well, in complete medium and incubated overnight. After the incubation period, the cell medium was removed and replaced with 100 µL of activated medium and incubated for a further 24 h at 37 °C, 5% CO_2_. The medium was then removed and 100 µL of complete medium containing 3–(4,5-dimethylthiazol-2-yl)–2,5-diphenyltetrazolium bromide (MTT) at 0.5 mg/mL was added to each well. Cells were incubated for 2 h at 37 °C. The medium was carefully discarded and 100 µL of dimethyl sulfoxide (DMSO) was added per well. The plate was then incubated at room temperature for 15 min under gentle stirring in order to dissolve MTT crystals. The absorbance of the solution was measured at 570 nm using a spectrophotometer (Safas, Monaco, Monaco). Cell viability was then calculated from a standard curve, generated under the same conditions, with control medium (w/o material) as 100%.

### 2.5. Antioxidant Activity

One specimen from each group (OB, OB@5%RS and OB@5%PY) was immersed in 10 mL of 2,2-diphenyl-1-picrylhydrazyl (DPPH) (10^−4^ mol/L in 70% ethanol). After 30 min and 2 h, 0.5 mL of each supernatant solution above the specimens was taken (after vigorous shaking) using a calibrated pipette. The absorption spectrum of each solution was measured at a wavelength range between 300 and 600 nm. The solution that was in contact with OB was used in the reference cell. Digital pictures of all DPPH solutions above the specimens were also captured after 5 min and 2 h of contact.

### 2.6. Release Kinetics of RS and of PY in Water and pH Measurement

Stock solutions of RS (0.1 mg/mL) and PY (0.1 mg/mL) were prepared and diluted gradually between 2 and 100 times with distilled water. Absorption spectra from each group were acquired using a double beam mc^2^ spectrophotometer (SAFAS, 98000 Monaco, Monaco) in order to establish a calibration curve allowing for the quantification of RS and PY release from the OB@RS and OB@PY sealers. The measurement cuvette was filled with a solution containing RS or PY, whereas the reference cuvette was filled with distilled water. One specimen from each material (OB, OB@RS and OB@PY) (containing 250 mg of each composite) was immersed in 15 mL of distilled water using a glass bottle. After 1, 3, 6, 24, 48 and 72 h, 0.5 mL of each supernatant solution was taken (after vigorous shaking). Subsequently, the absorption spectrum was measured (wavelength range between 200 and 700 nm). An absorption peak was observed at λ = 306 nm for the RS solutions and at λ = 267 nm for the PY solutions. The solution in contact with OB (control group) was used as reference. After each measurement, 0.5 mL of distilled water was added into each glass bottle in order to maintain a constant volume. pH measurements were performed at 24 ± 2 °C after incubation of the specimens in distilled water, under the same conditions as for the release experiment (3 h, 24 h and 72 h).

### 2.7. Solubility Evaluation

Three specimens from each tested material were prepared as reported in a previous study [3] and analyzed in accordance with the ISO standards 6876:2001. The specimens were weighed three times using a digital system (accuracy ± 0.0001 g) before different aging immersion periods (24 h and 7 days). After two immersion periods in 50 mL of distilled water at 37 °C, the specimens were removed from distilled water, gently washed with distilled water and dried at 37 °C for 24 h. Subsequently, the weight of each specimen was assessed three times and averaged to obtain the final weight. The solubility percentage of each tested material was determined from the difference in mass between the initial weight (before the immersion period in water) and the final weight [3].

### 2.8. Scanning Electron Microscopy (SEM) and Energy Dispersive X-ray (EDX) Analysis

Three specimens from each material group (OB, OB@5%RS and OB@5%PY) were created as described in Section 2.2 and immersed in phosphate-buffered saline (PBS10×, Dominique Dutscher, Bernolsheim, France) at 37 °C for 72 h and 7 days. After storage, all specimens were sputter-coated with gold-palladium (20/80) using a Hummer JR sputtering device (Technics, California, USA) and analyzed at a magnification of ×5000 for their chemical, mineral and morphological changes using an SEM-EDX (Quanta 250 FEG scanning electron microscope “FEI Company, Eindhoven, The Netherlands”; 10 kV acceleration voltage of the electrons). The weight percentages of the chemical elements were acquired from the outer surfaces of the specimens.

### 2.9. Water Sorption Tests, Roughness and Porosity Measurements

Five measurements were performed for each of the tested materials (OB, OB@5%PY and OB@5%RS), which were prepared as described in Section 2.2. A contact angle measurement device (Attention Theta, Biolin Scientific, Götenborg, Sweden) was used to measure the time of adsorption of an 8-microliter droplet of distilled water onto the surface of the tested materials. A video was recorded (10 frames per second) by a digital camera in order to track the profile of the water droplet and to calculate the contact angle of the water droplet at interval times.

The internal structures of OB, OB@5%RS and OB@5%PY were inspected in 3D by means of micro-computed X-ray tomography (µCT) (EasyTom 160 from RX Solutions, Chavanod, France). Imaging was conducted at a voltage of 45 kV and a current of 160 mA, using a micro-focused tube equipped with a tungsten filament. The source-to-detector distance (SDD) and the source-to-object distance (SOD) were adjusted in such ways to obtain a voxel size of around 2.3 µm. Volume reconstruction was performed with the software Xact64 (RX Solutions) after applying treatments such as geometrical corrections and ring artefact attenuation. Image treatment was performed with the Avizo software (ThermoFisher, Waltham, MA, USA) that enabled us to (i) de-noise the images with a median filter; (ii) segmentate the image intensity to reveal the objects of interest (here the pores); (iii) remove insignificant small objects (below a size of 10 pixels from the segmented 3D data); and (iv) determine the 3D geometrical aspects of the objects of interest (volume and equivalent diameter).

Atomic force microscopy (AFM) imaging was carried out in contact mode using the Bioscope Catalyst (Bruker Inc., Santa Barbara, CA, USA) equipped with MLCT cantilevers (0.7 N/m) (Bruker Inc., Santa Barbara, CA, USA). Images were obtained at a specific resolution (256 × 256 pixels) with a scan rate of 1 Hz (field of view of 10 × 10 µm). The roughness (Ra) was measured on three different representative regions of interest using the shareware software GWYDION.

### 2.10. Compressive Strength

Sixteen specimens were prepared from each test material and observed with an optical microscope at 10× magnification (Zumax medical, Suzhou New District, China) for the presence of voids or fractures. All specimens that presented such defects were discarded from the mechanical analysis. Half of the specimens were kept dry, while the other half were stored in water for 72 h. After storage, the specimens were tested using the uni-axial compression test in order to determine the stiffness of the cement and the maximum load before rupture. Such tests were performed using a universal electromechanical tensile Instron 3345 (Norwood, MA, USA) device instrumented with a 1 kN cell force (Class 0.5 following ISO 7500-1) and with a displacement sensor. The tests were performed at a constant crosshead speed of 0.5 mm/min. The compressive strength was calculated in megapascals (MPa) according to the following formula:

σc = 4P/πD^2^

where P is the recorded load during the test and D is the initial sample diameter.

### 2.11. Statistical Analysis

Statistical analysis was performed using SigmaPlot (release 11.2, Systat Software, Inc., San Jose, CA, USA). The Shapiro–Wilk test was used to verify the normality of the data in all groups. Analysis of the Variance (ANOVA) including a multiple comparison procedure (Holm-Sidak method) were used in order to determine whether significant differences existed in the compressive strength values, antibacterial activity, water sorption tests, roughness measurements and solubility evaluations between the different composites. In all tests, a statistical significance level of α = 0.05 was adopted.

## 3. Results

### 3.1. Antibacterial Activity

Generally speaking, the incorporation of PY into the sealer materials increased antibacterial activity against *Enterococcus faecalis*, whilst RS caused bacterial growth similar to the positive control.

#### 3.1.1. Agar Diffusion Tests

**Figure 1:**
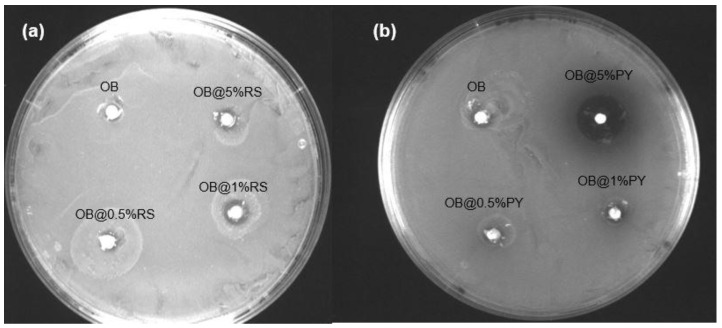
Agar diffusion tests with the different materials. (**a**) Control group (Obturys “OB”) and the sealer modified with different concentrations of resveratrol (OB@RS); (**b**) control group (OB) and the sealer modified with different concentrations of pyrogallol (OB@PY).

The OB sealer (control group) produced no inhibition zone on agar plates (Figure 1a,b). Conversely, OB@5%PY presented a large inhibition zone (5 ± 1 mm) (Figure 1b). OB@1%RS and OB@5%RS created only very small inhibition zones (1–1.5 mm). Note that the inhibition zones as well as part of the gel appeared brown in the presence of OB@5%PY as a result of the release of PY from the specimens (Figure 1b).

#### 3.1.2. Direct Contact Tests

**Figure 2:**
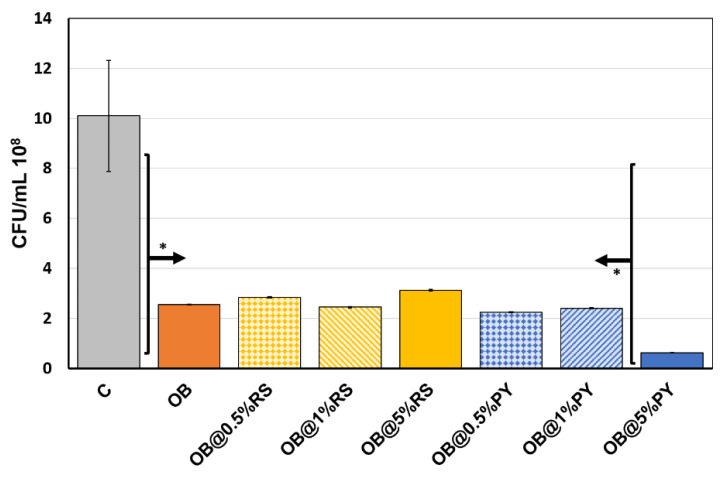
Number of colony forming units/mL of *E. faecalis* in the presence of sealer (OB) and sealer + different concentrations of RS/PY after 24 h of culture. C represents the control CFU experiment, without any material in each case. * *p* < 0.05.

The sealer (OB) caused significantly less bacterial growth than the control group (bacterial medium) (*p* < 0.05). The experimental OB@5%PY killed about 94% of *E. faecalis* bacteria after 24 h compared to the bacterial medium (Figure 2). In addition, OB@5%PY presented greater antibacterial activity than OB (<0.05). However, no differences were found between OB and OB@0.5%RS/PY, OB@1%RS/PY and OB@5%RS at 24 h; they all killed between 70% and 78% of the bacteria (Figure 2). However, it was evident that 5%PY was by far much more efficient than 5%RS against *E. faecalis*.

### 3.2. Antioxidant Activity

**Figure 3:**
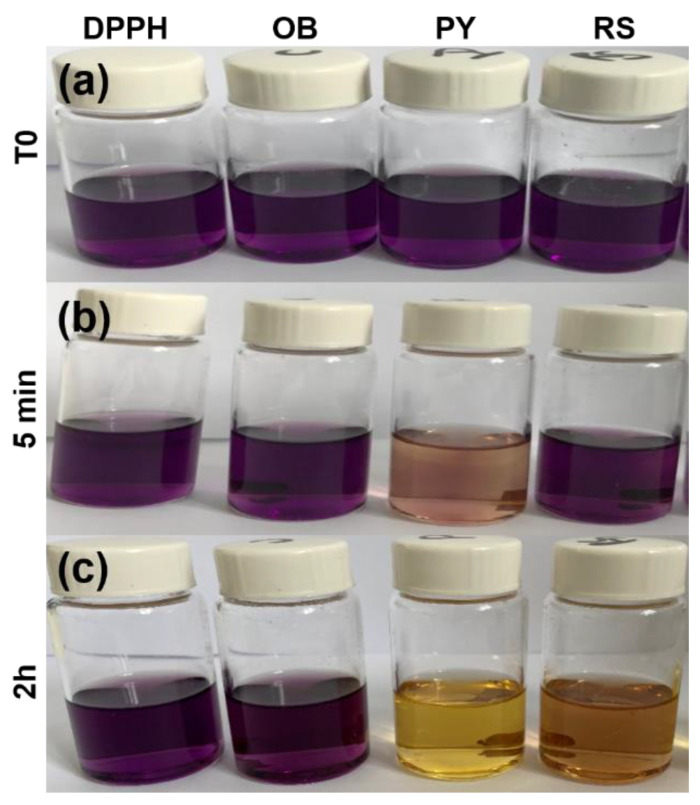
Digital pictures of color changes undergone by a 2,2-diphenyl-1-picrylhydrazyl (DPPH) solution (10^−4^ mol/L in 70% ethanol); (**a**) before (upper row, T0); (**b**) after 5 min; and (**c**) after 2 h of contact with the different composites.

As expected, the control group (OB) had no effect on the color of the DPPH solution, hence there was no antioxidant activity (Figure 3). However, incorporation of either PY or RS provided this additional property to the experimental sealers tested in this study (Figure 3). After 5 min and 2 h of contact between the DPPH solution and tested materials, the solution in contact with the PY group appeared much clearer than the solution in contact with the RS group (Figure 3, Table 2). Using an extinction coefficient of 1.09 × 10^4^ L·mol^−1^·cm^−1^ for DPPH at λ = 525 nm [22], we calculate from the absorbance decrease values provided in Table 2 that 53% and 43% of DPPH are quenched after 30 min of contact with OB@5%PY and with OB@5%RS, respectively. One PY and one RS molecule are equivalent to three TROLOX molecules from an antioxidant point of view [22].

### 3.3. Cytotoxicity

Cytotoxicities of leachable components from OB, OB@5%RS and OB@5%PY were evaluated at 24 h and it was observed that OB and OB@5%RS have good cell viabilities, (92.79 ± 6.18)% and (87.93 ± 4.89)%, respectively. In contrast, at 24 h it was observed that activated media from OB@5%PY showed acute toxicity with 0% cell viability.

### 3.4. Release Kinetics of RS and of PY in Water and pH Measurement of Water in Contact with the Composites

**Figure 4:**
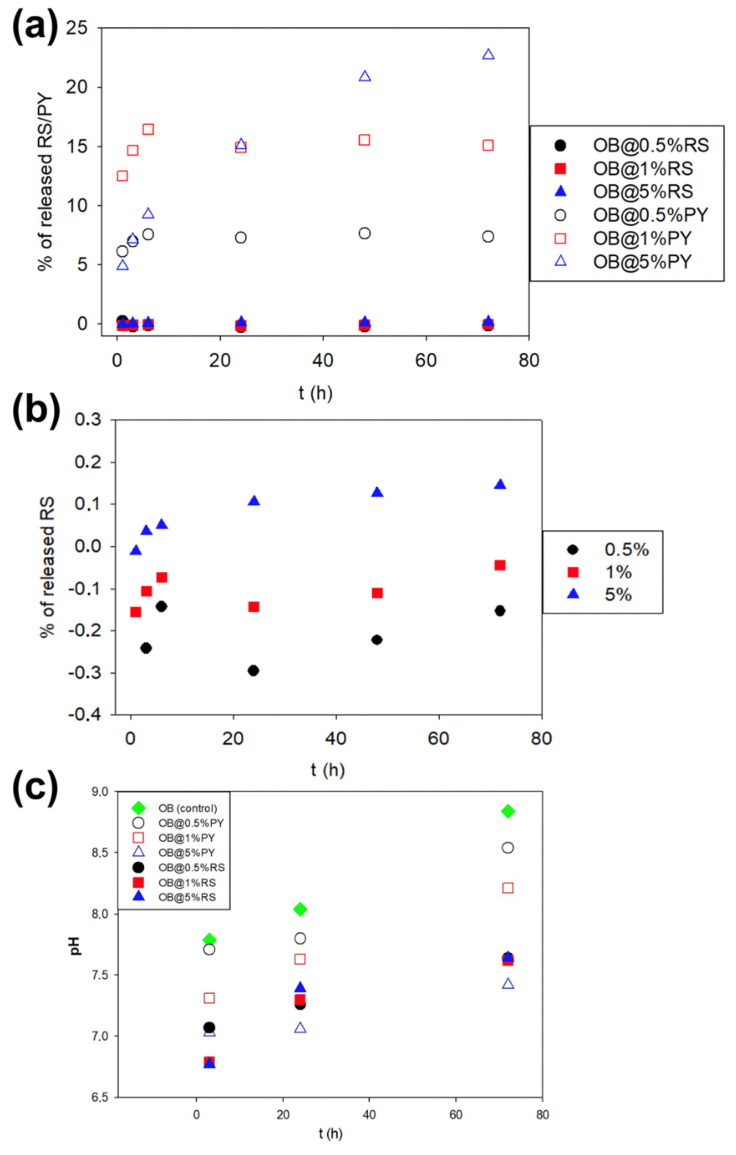
(**a**) Percentages of RS and PY released from the different composites versus time with respect to different initial mass fractions in the composites; (**b**) percentages of RS released from the different composites versus time with respect to the different initial mass fractions in the composites; (**c**) pH changes with time of water put in contact with sealer sample (control) and sealer modified with different concentrations of PY and/or RS as shown in the inset.

A maximum of 22.5% of the initial PY was released after 72 h (Figure 4) for OB@5%PY. In contrast, the maximum release of OB@RS was 0.2%, which was reached in the case of the addition of 5% RS (Figure 4a). These results showed that the higher the PY concentration, the higher the concentration of PY released in water (Figure 4). The release of PY and RS induced a slight decrease in the pH of water in contact with the different materials. After 72 h, such a reduction in pH was more pronounced in the case of OB@5%PY compared to the other tested materials (Figure 4b).

### 3.5. Solubility Evaluation

**Figure 5:**
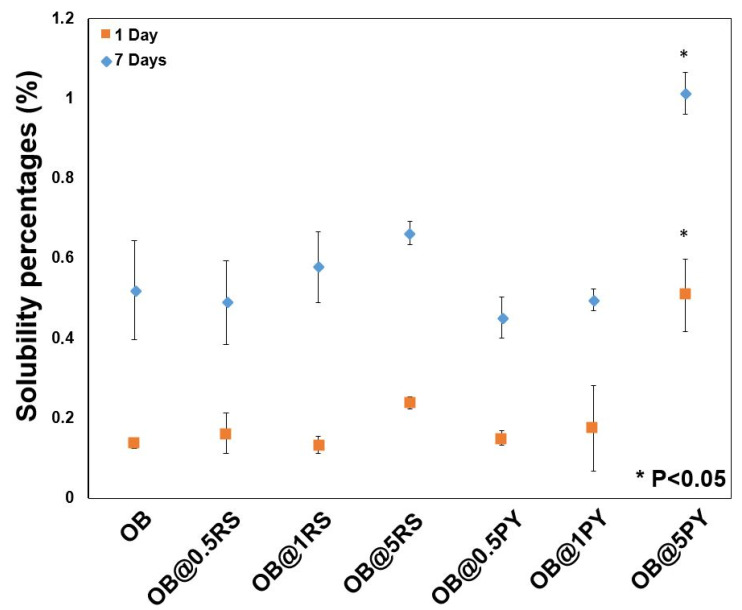
Solubility percentages (wt.%) (*n* = 3) of OB and OB modified with different concentrations of RS and PY in distilled water at 37 °C after 1 and 7 days. * *p* < 0.05.

The results of solubility percentages (wt.%) of the tested composites after 1 and 7 days are presented in Figure 5. None of the solubility percentages exceeded 1.1% after 7 days. OB@5%PY demonstrated significantly higher solubility percentages for both testing periods compared to all the other tested materials (*p* < 0.05).

### 3.6. Scanning Electron Microscopy (SEM) and Energy Dispersive X-ray (EDX) Analysis

**Figure 6:**
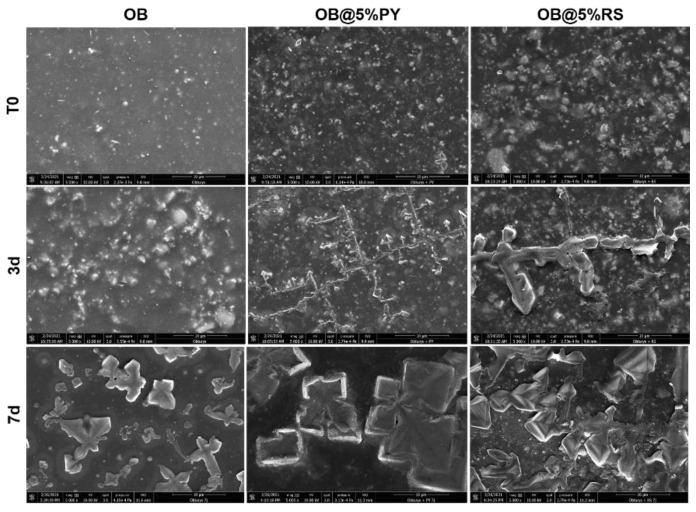
Representative scanning electron microscopy images at 5000× magnification. The morphologies of OB, OB@5%RS and OB@5%PY surfaces were analyzed in dry condition (T0); and after 3 and 7 days in PBS at 37 °C.

The results of the tested materials’ surfaces after immersion in PBS are depicted in Figure 6 and Table 3. Larger crystallites were observed on OB@PY/RS surfaces after 7 d compared to those on the OB surface. The chemical analyses of these surfaces determined by EDX analysis showed a remarkable decrease in the carbon content of the large crystallites in the RS and PY specimens after 7 d. The different chemical compositions are provided in Table 3.

### 3.7. Water Sorption Tests and Roughness

**Figure 7:**
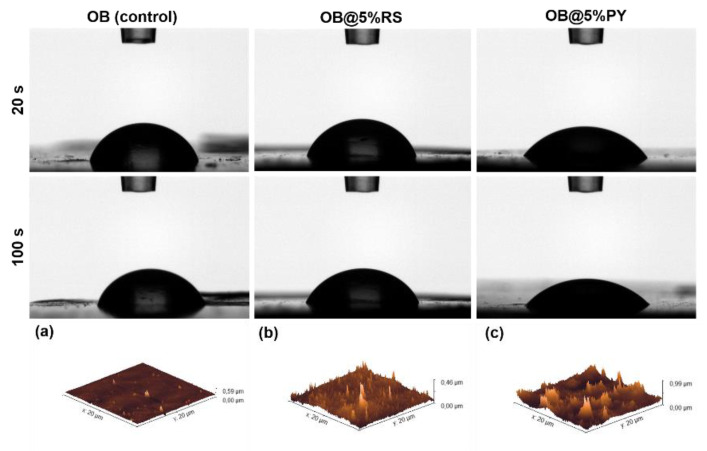
Contact angles of a water droplet (initial volume of 8 µL) deposited onto different composite surfaces (OB, OB@5%RS and OB@5%PY) after 20 and 100 s post deposition. AFM micrographs (20 µm × 20 µm) of the different composite surfaces (**a**) OB, (**b**) OB@5%RS and (**c**) OB@5%PY.

A further significant influence of the addition of PY (5%) into the epoxy-resin structure was a resulting increase in its hydrophilicity (Figure 7). Contact angles of (58 ± 1)° and (46 ± 2)° for OB@5%PY were detected after 20 s and 100 s, respectively. These were significantly lower than the respective contact angles observed in OB (74 ± 4°, 66 ± 5°) and OB@5%RS (68 ± 5°, 62 ± 5°), *p* < 0.05. No significant differences were found between OB and OB@5%RS (*p* > 0.05). All tested materials analyzed using AFM displayed rough surfaces (Figure 7a–c). However, a rougher surface was observed in OB@5%PY (69.45 ± 17.3 nm) compared to OB control (11.11 ± 5.9 nm, *p* < 0.001) and OB@5%RS (30.27 ± 10.27 nm, *p* = 0.003).

### 3.8. Compressive Strength and Porosity

**Figure 8:**
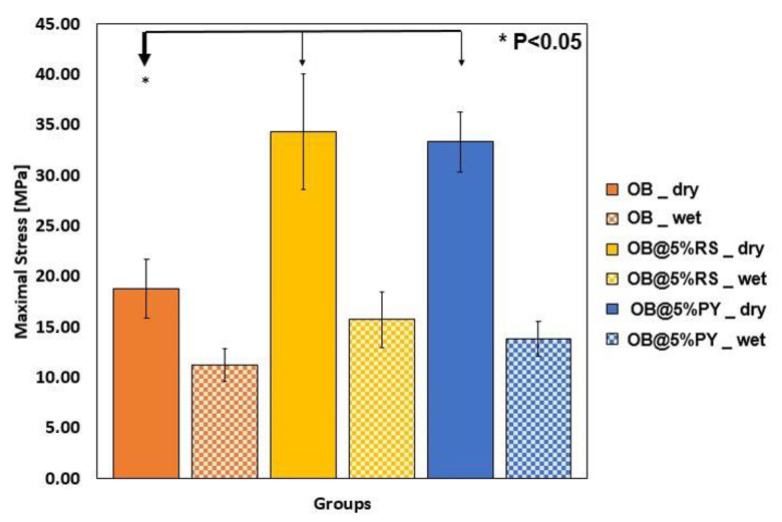
Evolution of maximal stress under compression for OB, OB@5%RS and OB@5%PY in dry conditions; and after immersion in water for 72 h. * *p* < 0.05 bold arrow.

In Figure 8 it is possible to compare the compressive strength values of the tested materials. There were significant differences between OB (18.78 ± 2.91 MPa) and both OB@5%RS (34.30 ± 5.71 MPa) and OB@5%PY (33.34 ± 2.98) in dry conditions (*p* < 0.05). However, all compressive strength values decreased after 72 h of storage in water at 37 °C (Figure 8). No significant differences were found among the compression values of all tested composites in wet conditions (*p* > 0.05).

**Figure 9:**
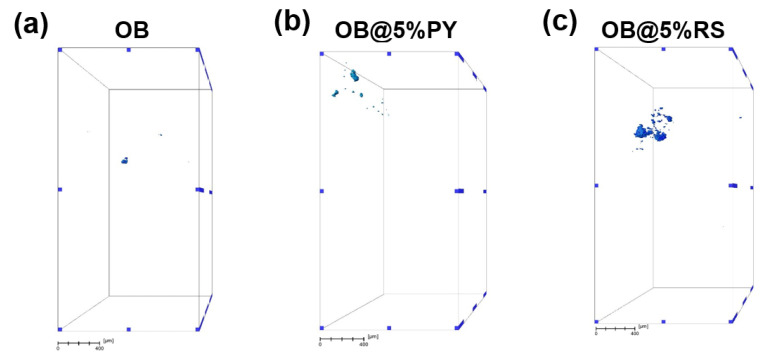
Micro-computed X-ray tomography analysis (scale bar of 400 µm). Volume rendering of the segmented pores in (**a**) OB (unmodified sealer); (**b**) OB@5%PY (sealer doped with 5%PY); and (**c**) OB@5%RS (sealer doped with 5%RS).

The pore average equivalent diameters and volume percentages were evaluated for the different materials using µCT. No notable differences were found between the unmodified sealer when compared to the sealer doped with RS or PY (Figure 9, Table 4). Therefore, the three cases exhibited quite similar pore characteristics.

## 4. Discussion

To the best of our knowledge, this is the first study to incorporate pyrogallol and/or resveratrol into an epoxy-resin endodontic sealer in order to modify its biological, mechanical and physicochemical properties. Indeed, the general hypothesis of this study was that the addition of such polyphenols into epoxy resin-based sealers would have provided an enhancement to some biological and mechanical qualities. In the current study, incorporation of the tested polyphenols was performed into the base part (Table 1) of the epoxy resin-based sealer. We believe that since the catalyst part of the epoxy resin contains amines, oxidation of PY could have activated the polymerization and/or reduced beneficial effects of the phenolic compounds via formation of covalent bonds with the amines [23]. One of the first outcomes obtained in this study was that the control (free of polyphenols) sealer had some antibacterial effects against *E. faecalis* after 24 h. Ruiz- Linares et al. [24] analyzed an epoxy resin-based sealer (AH-Plus, Dentsply Sirona, Konstanz, Germany) against *E. faecalis* and they showed that such material could induce some antibacterial effects as a result of the release of formaldehyde and/or other toxic non-polymerized components, such as amines or epoxy monomers.

However, the incorporation of 5 wt%PY into the epoxy-resin sealer promoted greater antibacterial effects against *E. faecalis* compared to other experimental sealers doped with different percentages of RS. Both antibacterial tests (DCT and ADT) showed great antibacterial efficacy for the sealer doped with 5%PY as well as a peculiar brown-colored zone in the agar (Figure 1b). The brown color observed during the study with the experimental material was probably induced by oxidation of the pyrogallol in the bacterial medium [16,21]. In accordance with such a hypothesis, increased and more rapid antioxidant activity were observed for the OB@5%PY sealer when compared to specimens in the OB@5%RS group. Our hypothesis is that such findings may be principally associated with a relatively significant release of PY from the experimental materials, in comparison to release of RS (Figure 4a). Moreover, the significant release of PY may be a consequence of its smaller molecular weight compared to that of RS. From a chemical point of view, PY has three hydroxyl groups on the benzene ring, whilst the RS molecule has two benzene rings with three hydroxyl groups. It is possible that the different positions of the hydroxyl groups may have affected antibacterial activity of the experimental materials, exactly the same as that described by Cueva et al. [25]. Indeed, those authors reported that the numbers and positions of substitutions in the benzene rings of the phenolic acids, along with the saturated side-chain lengths, influenced antimicrobial properties of the phenolic acids against different microorganisms. The antibacterial effects of PY incorporated into dental adhesive (adhesive-resin with acidic function monomers) against *Streptococcus mutans* and its effects against Staphylococcus aureus when incorporated into plaster of Paris were previously reported [16,21]. Moreover, the antibacterial properties of resveratrol against various *bacteria*, including *E. faecalis*, were also reported in a previous study [26].

The antioxidant activity of OB doped with the tested polyphenols was evaluated using the DPPH discoloration method [16]. OB@5%PY presented increased and more rapid antioxidant activity than OB@5%RS. As stated before, these findings could be related also to the greater release of PY from the tested materials. Atalayin et al. [13], reported that RS reduced DNA damage and reactive oxygen species (ROS) produced by different dental resin-adhesive systems. Platzer et al. [27] reported significant antioxidant activity of PY associated with its structure and number of OH groups. Our study showed that resveratrol has good biocompatibility when it is incorporated into an epoxy-resin endodontic sealer, whilst PY can exert a cytotoxic effect on fibroblasts (NIH3T3). However, the protective effects of RS against ROS production and the decrease in cell viability were previously reported [13]. In contrast, the significant PY release from tested materials induced a negative effect on cell viability (Figure 3). This is in accordance with the observations of Park [28], who reported that PY was relatively toxic for human pulmonary fibroblasts (HPF), and induced cell death and a dose-dependent decrease in cell growth. Our study has also shown that the liberation of PY in water induced a slight decrease in pH (Figure 4b). Similar observations were reported in previous studies [16,21]. Subsequent to the evaluation of the biological properties of the experimental epoxy-resin sealer doped with PY or RS, their solubilities were also analyzed in accordance with the ISO standards 6876:2012; the solubility of dental sealer should not exceed 3% mass after 24 h in water [3]. It is known that high solubility may create pathways for microorganism infiltration into the root canal system [3]. The epoxy resin doped with 5%PY demonstrated the highest solubility compared to other tested materials. However, this solubility did not exceed 3% mass after 24 h and 7 d. Such findings may be correlated to the significant release of PY from the experimental sealers tested in this study. SEM was used to observe morphological changes induced by the incorporation of PY or RS into the experimental sealers. Larger crystallites were observed on the surfaces of OB@5%PY and of OB@5%RS rather than OB (unmodified epoxy-resin) after an immersion period of 7 days in PBS (at 37 °C).

Different polyphenols have been used in dental applications in order to evoke mineral precipitation and remineralization [10]. For instance, grape seed extracts were used in order to induce mineral precipitation in dentin [29]. Liu et al. [30] demonstrated that resveratrol could reduce tooth movement and root resorption during orthodontic therapy. Furthermore, gallic acid facilitated the participation of hydroxyapatite crystallites with specific particle size [31]. The sorption test applied with the different experimental materials was performed using a contact angle method. The profile of an 8-microliter droplet of distilled water on each composite surface was analyzed and the OB@5%PY sealer demonstrated greater wettability (lower contact angle) compared to OB and OB@5%RS. These findings suggest that OB@5%PY may have a greater surface energy [32,33]. Moreover, OB@5%PY presented a rougher surface during AFM analysis compared to OB and OB@5%RS. The greater surface roughness may in part explain the wettability results obtained with some specific experimental materials tested in this study. Indeed, one of the main factors that may assign the contact angle measurements and wettability evaluations is the surface roughness [33]. In accordance with Wenzel or Cassie-Baxter [33], chemical composition of the surface can also play an important role in contact angle measurements. Therefore, we can affirm that the OB@5%PY sealer had both a rougher and more hydrophilic surface than other composites. Zuo et al. [34] reported that PY could be applied in polymer materials in order to fabricate hydrophilic coatings. The mechanical properties of endodontic sealers are very important to strengthen treated root canals as well as to increase resistance to displacement of the gutta-percha cone during and after its placement [3,35]. The results of this study showed that the addition of both polyphenols in an epoxy-resin sealer significantly increased its compression stress values in dry conditions compared to the unmodified control sealer. However, no notable differences were observed for the pore size/volume between different materials tested in this study. The higher compressive strength of OB@PY/RS compared to the control group may be explained by the hypothesis that these polyphenols play the role as a sort of bonding agent between sealer elements. Moreover, it has been demonstrated that the presence of a polyphenol, TA, in mineral trioxide aggregate could increase a sealer’s compressive strength [4]. Such an outcome may be related to the ability of such molecules to adsorb on all kinds of surfaces [36] and to ultimately favor interparticular interactions. After immersion in water, both experimental materials showed lower compressive strength values compared to the same materials tested in dry conditions. It is hypothesized that such outcomes may be a result of the liberation of polyphenols in water; this may have degraded bonding links that were probably present in dry conditions. However, no statistical differences were found between the unmodified sealers in dry or wet conditions when compared to OB@PY/RS in wet conditions. Therefore, the compressive strengths of sealers modified with the tested polyphenol, after immersion in water, yielded similar results to the unmodified sealer. In accordance, Kharouf et al. [4] reported an increase in compressive strength of mineral trioxide aggregate (MTA) doped with tannic acid in dry conditions. However, they also found that MTA doped with tannic acid presented weaker mechanical properties in wet conditions. The current study showed both positive and negative outcomes when pyrogallol or resveratrol were incorporated into epoxy-resin sealers. For instance, mechanical properties and antioxidant activity of the epoxy-resin dental sealer were enhanced by incorporating RS and PY into its compositions. The material doped with pyrogallol was more efficient in terms of antibacterial activity against *E. faecalis* than the experimental sealer doped with resveratrol or the control unmodified resin. However, pyrogallol displayed high toxicity towards fibroblasts, and such cytotoxicity marred other favorable qualities (antibacterial and mechanical properties) produced from pyrogallol incorporation into endodontic sealers. In contrast, resveratrol presented better biocompatibility despite its limited antibacterial activity when compared to the pyrogallol group. For this reason, resveratrol may be considered as a suitable choice for incorporation into endodontic sealers with its important mechanical and antioxidant effects compared to unmodified sealers. Antioxidant activity introduced into an endodontic sealer could be useful in preventing harmful effects of oxidative stress and in stabilizing free radicals, thereby protecting cells from damage [37]. As mentioned in Section 2.2, the addition of both polyphenols was limited to 5% (wt.%) to avoid inhomogeneity within the materials resulting from phase separation. Therefore, our future studies will be focused on alternative strategies to incorporate more RS into an epoxy-resin sealer in order to enhance its antibacterial properties without affecting its cellular viability rate. However, the concentration of microorganisms found in root canal systems may be lower compared to those used in our DCT test. Hence, lower concentrations of PY or RS than those used in this in vitro study may be also suitable for real clinical scenarios. In addition, further investigations are required to analyze chemical interactions between the epoxy-resin sealer and polyphenols, and their effects on bond strength to root canal dentin.

## 5. Conclusions

Within the limitations of the present study, the addition of pyrogallol and resveratrol to an epoxy-resin dental sealer improved mechanical and biological properties. However, pyrogallol seems to have greater antibacterial and antioxidant activities than resveratrol, whereas the latter has fewer cytotoxic effects.

## Figures and Tables

**Table 1:** Endodontic sealer and polyphenols: chemical compositions and references.

Materials	Reference	Chemical Composition
Obturys (OB)	OBAX1-5	Base: DGEBA, zirconium oxide, bismuth oxychloride, ytterbium oxide, fumed silica Catalyst: diamine, zirconium oxide, bismuth oxychloride, ytterbium oxide, fumed silica, silane quaternary ammonium salt
Pyrogallol (PY)	P0381-250G	C_6_H_3_(OH)_3_ 1,2,3-Trihydroxybenzene
Resveratrol (RS)	R5010-500MG	C_14_H_12_O_3_ 3,5,4′-trihydroxy-*trans*-stilbene

**Table 2:** Antioxidant activity, as measured by a reduction in absorbance at 525 nm corresponding to a discoloration of 2,2-diphenyl-1-picrylhydrazyl (DPPH) solutions in contact with OB, OB@5%RS and OB@5%PY composites.

	30 min	2 h
PY (525 nm)	−0.58 ± 0.02	−0.54 ± 0.01
RS (525 nm)	−0.47 ± 0.01	−0.45 ± 0.004

**Table 3:** Relative compositions of the experimental sealers as measured by means of EDX at T0 and after 7 days of immersion in PBS.

	Elements	T0	T7 Days
**Obturys (OB)**	C (%)	70 ± 2	66 ± 4.5
	O (%)	19 ± 1	17 ± 1.6
	Zr (%)	7.5 ± 2	5 ± 2.3
	Si (%)	1.5 ± 0.2	1 ± 0.4
	Cl (%)	1 ± 0.1	6 ± 2
	Na (%)	0	5 ± 1.8
**OB@5%RS**	C (%)	63 ± 1.5	53 ± 4
	O (%)	18 ± 0.8	15 ± 2
	Zr (%)	14 ± 1	11 ± 2
	Si (%)	2.2 ± 0.3	1.6 ± 0.4
	Cl (%)	1.6 ± 0.8	10 ± 4
	Na (%)	0	8.5 ± 3.6
**OB@5%PY**	C (%)	62 ± 2.5	48 ± 11
	O (%)	20 ± 0.2	12 ± 2.2
	Zr (%)	13 ± 3	7 ± 3
	Si (%)	2.5 ± 0.4	1 ± 0.5
	Cl (%)	1.7 ± 0.8	17 ± 7
	Na (%)	0	14 ± 5

**Table 4:** Pore characteristics of OB, OB@5%RS and OB@5%PY as calculated from µCT imaging.

Group	Pore Average Equivalent Diameter (µm)	Pore Volume Density (%)
OB	15.1	1.2 × 10^−3^
OB@5%PY	14.0	5.1 × 10^−3^
OB@5%RS	19.0	2.6 × 10^−2^

## Data Availability

The data presented in this study are available on request from the corresponding author. The data are not publicly available due to the reason that a small part of these data are shared with a private company.
